# Pressure Pain Threshold of the Upper Trapezius Trigger Point: A Systematic Review with Meta-Analysis of Baseline Values and Their Modification after Physical Therapy

**DOI:** 10.3390/jcm11237243

**Published:** 2022-12-06

**Authors:** Tommaso Geri, Alice Botticchio, Giacomo Rossettini, Sanaz Pournajaf, Leonardo Pellicciari, Stefano Di Antonio, Matteo Castaldo

**Affiliations:** 1 Independent Researcher, 51100 Pistoia, Italy; 2 Independent Researcher, 25121 Brescia, Italy; 3 School of Physiotherapy, University of Verona, 37134 Verona, Italy; 4 Neurorehabilitation Research Laboratory, Department of Neurological and Rehabilitation Sciences, IRCCS San Raffaele Roma, 00163 Rome, Italy; 5 IRCCS Istituto delle Scienze Neurologiche di Bologna, 40126 Bologna, Italy; 6 Center for Pain and Neuroplasticity (CNAP), Department of Health Science and Technology, School of Medicine, Aalborg University, 9220 Aalborg, Denmark; 7 Department of Neuroscience, Rehabilitation, Ophthalmology, Genetics and Maternal Child Health, University of Genoa, 16132 Genoa, Italy; 8 Poliambulatorio FisioCare, 16035 Rapallo, Italy; 9 Sport Physiotherapy, University of Siena, 53100 Siena, Italy; 10 Department of Physical Therapy, Poliambulatorio Fisiocenter, 43044 Collecchio, Italy

**Keywords:** trigger points, physical therapies modalities, rehabilitation

## Abstract

Background: Myofascial trigger points (TrP) are diagnosed upon the presence of clinical signs among which hypersensitivity is considered one of the most important. The detection of the pressure pain threshold (PPT) is used to quantify the degree of hypersensitivity. However, there is a lack of normative data about how hypersensitive a TrP is. Therefore, the objective was to quantify the PPT for myofascial TrP in the upper trapezius muscle and its modification after manual or instrumental physical therapy interventions. Methods: A systematic review and meta-analysis were conducted among three databases (MEDLINE, Cochrane Library, and PEDro). Two independent reviewers conducted the electronic search and assessed the methodological quality of the included studies. Results: Eleven studies with a high-risk bias indicated that the PPT at TrP sites was 105.11 kPa lower (95% CI: −148.93; −61.28) at active TrP sites (Chi-squared = 1.07, df = 1 (*p* = 0.30), I^2^ = 7%) compared to the PPT of the upper trapezius muscles of healthy subjects. In addition, the PPT of TrP was also lower than the reference values coming from the pain-free population. Moreover, the PPT increased after both manual and instrumental treatment by 28.36 kPa (95% CI: 10.75; 45.96) and 75.49 kPa (95% CI: 18.02; 132.95), respectively. Conclusions: The results of the present study show that TrP has a decreased PPT when compared to healthy muscles and that physical therapy may increase the PPT. However, the clinical relevance of this decreased PPT needs to be further elucidated. Further, the high risk of bias in all the retrieved studies undermines the validity of the results.

## 1. Introduction

A trigger point (TrP) is defined as a hypersensitive spot within a contracted muscle fiber, that is painful to compress, can induce referred pain, and can generate autonomic phenomena [1]. Other symptoms usually reported are muscle stiffness, spasms, and limitations in movement of adjacent joints [2]. Several musculoskeletal pain syndromes are thought to be associated with TrP and are considered under the umbrella term “myofascial pain syndromes” [1], whose prevalence is generally reputed to be high [3,4]. An active TrP is defined when its palpation can reproduce the familiar pain or referred pain pattern of the patient, either present or past. In contrast, a latent TrP is defined when the somatosensory sensations evoked during palpation are not related to the patient’s symptoms [5]. The nociceptive afference arising from both latent and active TrPs is thought to increase the central excitability of the nervous system, causing peripheral [6] or central sensitization [7,8], in which the alteration of the dorsal root ganglion and an expansion or new formation of the receptive fields are considered responsible for the referred sensation evoked during palpation [9].

Manual muscle palpation constitutes an important procedure for the clinical assessment of the TrP, whose diagnosis lacks a proper gold standard. According to experts’ opinion, the three main clinical findings to diagnose a TrP are the detection of a taut band, the detection of a hypersensitive spot inside, and the elicitation of referred pain [5]. The presence of a local twitch response was an additional criterion reported in a systematic review that also found disagreement on which are the most important criteria to be used [10]. For this reason, studies on the reliability of this palpatory examination showing poor to moderate intra and inter-reliability undermine its use in clinical practice [11,12]. The limits in quantifying the extent of hypersensitivity intrinsic to palpatory examination are overcome with the use of pressure algometry, a device that, by applying increasing force over a limited constant surface, allows the quantification of the minimum pressure, reported in kg/cm^2^ or kilopascal (kPa), and is able to induce pain or discomfort, indicated as the Pain Pressure Threshold (PPT). A reduction in PPTs is merely interpreted as the increased sensitization of the painful body part, or of body parts far from the painful area that reflect, respectively, the degree of peripheral and/or central sensitization of the pain pathways. Indeed, it is important to remember that a lowered PPT may be a proxy of central sensitization if it is also found in healthy, pain-free areas [7,13]. Otherwise, if PPTs are lowered only in the symptomatic area, they are considered as the expression of peripheral sensitization [6].

The detection of a PPT specific to TrPshas been shown as a reliable procedure for the diagnosis of the TrPs themselves [14,15], and normative values for healthy subjects have been provided [16]. However, for muscles with TrP, there are a lack of normative PPT values that may inform decisions (more than manual muscle palpation) on the presence of a hypersensitive spot when diagnosing a TrP. For example, the measurement of the PPT is recommended to establish the extent of increased pain sensitivity in patients with headaches [15,17]. The PPT is considered clinically meaningful when its value is around 20% less than the PPT of healthy subjects for the same muscle [16]. Although no differences between dominant and non-dominant arms are reported [15], lowered thresholds are usually found in women compared to men, in older adults compared to younger adults and in lower limb/trunk muscles compared to upper quadrant muscles [16]. In clinical practice the PPT of the affected muscle is usually compared with the contralateral healthy side or with the lower limb muscles for patients with a pain condition of the upper quadrant. However, it is worth noting that when central sensitization is suspected, such as when multiple TrPs are found, lowered PPTs are also retrieved in the contralateral healthy side [7,18,19]. Neziri et al. [20] and Waller et al. [21] have suggested that values at the 5th and 95th percentile of the PPT distribution in a pain-free population indicate hyper and hyposensitivity thresholds, respectively. Referring to these absolute reference values would be helpful when dealing with patients with central sensitization.

An increase in the PPT has been proposed as a suitable parameter for the efficacy of treatment targeting TrPs, indicating less mechanical sensitivity over the TrP region [15,17]. Indeed, several studies on treatment efficacy have been conducted that measure the PPT at the TrP site before and after an intervention. Among these are dry needling [22,23], botulinum toxin [24], ischemic compression therapy [25,26], Kinesio taping [27], as well as lidocaine patches [28], exercises, and massage [29]. Although any of these treatments may be claimed to be effective in managing TrPs, a normative value of a clinically meaningful amount of pre-post difference in the PPT following whatever intervention has not been established yet. This kind of value may inform the clinical effectiveness of the intervention beyond its statistical significance. Among the numerous studies on the PPT, many have been conducted on the upper trapezius muscle, which therefore represents a suitable model given its high involvement in many musculoskeletal pain syndromes of the upper quadrant and its anatomical position that allows accessibility to both manual and instrumental assessment and treatment. The purpose of this systematic review and meta-analysis is to evaluate whether upper trapezius muscles with TrPs have a different PPT when compared to healthy muscles and whether they resemble the suggested hypersensitivity threshold [20,21]. Furthermore, it will be analyzed whether physical therapy treatment of a TrP is able to influence the PPT of the treated muscle.

## 2. Materials and Methods

### 2.1. Protocol and Registration

The protocol of this systematic revision was prospectively registered at PROSPERO (https://www.crd.york.ac.uk/prospero/ accessed on 3 November 2022) with the number CRD42020152611.

### 2.2. Eligibility Criteria

Type of studies: the study types considered were non-randomized and randomized controlled trials (RCT) based on manual or instrumental physical therapy treatments, except for case reports and case series.

Type of participants: studies were included when participants were older than 18 and presented an active or latent TrP in the upper trapezius muscle or no TrP in the same muscle in the case of healthy subjects recruited as controls. Studies were excluded when participants had any of the following conditions: the presence of comorbidities due to medical disease (neurological, rheumatic, oncology, cardiac, or metabolic dysfunctions) or previous surgical interventions in the examined area.

Type of interventions: the measurement of the PPT was made with both electrical and manual pressure algometry using kg/cm^2^ or kPa as the unit of measure or providing values allowing transformation into kPa.

Type of comparators: acceptable comparators were the same muscle in a healthy group recruited in the same study.

Type of outcomes: the primary outcome was the PPT difference between an active or latent TrP and healthy control muscles. Another primary outcome was the post-treatment PPT values between the intervention and placebo control group. The PPT was converted into kPa according to the unit of measure used in the study and the dimension of the probe used.

### 2.3. Search Strategy

The search was performed using the databases MEDLINE (through the search engine PubMed), Cochrane, and PEDro, looking for online publications until 31 August 2020. The search terms used were myofascial pain, trigger point or trigger points, pressure pain threshold, algometry or algometer. Relevant articles were screened for additional RCTs to consider. The full strategy is reported in Appendix A.

### 2.4. Study Selection

Two authors (A.B., S.D.A.) independently searched the databases to identify appropriate records to screen, applying the eligibility criteria. When the screening process ended, the full text of the identified records was retrieved and assessed for eligibility in the qualitative/quantitative synthesis.

Any disagreement was resolved by consensus; if no consensus was reached, a third reviewer (T.G.) made the final decision. The inter-rater agreement of the screening and of the eligibility processes before consensus were expressed using a percentage agreement and Cohen’s kappa [30].

### 2.5. Data Collection

Two independent authors (A.B., S.D.A.) manually extracted data from the included studies, filling a pre-formatted table that included data about population samples, type of myofascial disorder, nature of TrPs (active or latent), analyzed muscles, type of algometer used and, for RCTs, treatment conducted. Since PPT values have already been shown to be different between the sexes, sex-disaggregated data were not calculated in the present review. Any disagreement was resolved by consensus; if no consensus was reached, a third reviewer (T.G.) made the final decision.

### 2.6. Risk of Bias (RoB) Assessment

The Cochrane Risk of Bias tool version 2.0 (RoB2.0) [30] was used to assess the internal validity of the included RCTs. In addition, non-randomized clinical trials were assessed using the Risk Of Bias In Non-randomized Studies of Interventions (ROBINS-I) tool [31]. For RoB2.0, the domains randomization process, deviations from the intended intervention, missing outcome data, measurement of the outcome, and results reporting were evaluated to obtain for each study an overall risk of bias judgement that ranged from low—when all domains have a low risk of bias—to high—when the study has at least one domain with high-risk bias or multiple domains showing biasing concerns. For ROBINS-I, the domains evaluated were confounding bias, selection bias, classification of intervention bias, missing data, measurement of outcome, and selection of reported result. Two independent authors (A.B., L.P.) assessed the included studies, and a third reviewer (TG) made the final decision when consensus could not resolve the disagreement. The inter-rater agreement of the assessment of the risk of bias before consensus was calculated using percentage agreement and Cohen’s kappa [30].

### 2.7. Analysis and Synthesis of Results

The PPT was analyzed using the pooled mean difference (MD). The variance was expressed with 95% confidence intervals (95% CI). As our interest was understanding the treatment effect against a placebo control, the PPT values derived from different intervention arms of the same RCT study [32,33,34] were merged using well-established methods [35]. A global PPT value for active and latent TrPs was obtained by calculating the weighted mean and SD using the values reported in individual studies. The obtained values were compared with the weighted mean of values from two studies reporting a PPT on the upper trapezius muscle in the general population [20,21]. The comparison was made with a one-sample t-test. Alpha was 0.05.

The outcome measures from the individual trials were combined through meta-analysis where possible using the random-effects models described by DerSimonian and Laird [36] as some heterogeneity of population and treatments would be expected among interventions.

Heterogeneity was analyzed by means of the I^2^ statistic and the Chi^2^ test. A *p*-value lower than 0.1 indicated the presence of a statistically significant heterogeneity for the Chi^2^ test [36]. The degree of heterogeneity was expressed with the percentage of I^2^. Percentage values of 25, 50, and 75% indicated a low, moderate, and high degree of heterogeneity, respectively [36]. If a study did not provide usable summary measures for an outcome, it was included in the review but excluded from the meta-analysis, e.g., Gemmell et al. [37] and Kavadar et al. [38]. For the included studies, the numbers lost to follow-up in each group and the reasons for attrition were recorded. For missing data, the similarity of the group was evaluated, then the corresponding authors of the included studies were contacted (e.g., by emailing or writing to the corresponding author), and if no information was provided we conducted analyses using only the available data (e.g., we did not impute missing data). Cohen’s kappa and percentage agreement (PA) were judged as acceptable when higher than 0.6 and 80%, respectively [31]. Analysis was performed using Revman 5.0 [39] and R software [40] with Hmisc package v 4.4-0 [41].

The reporting of this study has been performed in accordance with the Preferred Reporting Items for Systematic Review and Meta-Analysis (PRISMA) statement [42].

### 2.8. Level of Evidence

The overall quality of evidence was evaluated using the grading of recommendations assessment, development, and evaluation approach (GRADE) for the main outcome based on the methodological quality of the included trials [43]. In addition, the quality of the evidence was evaluated using the software GRADEPro GDT [44], which assessed the risk of bias, inconsistency, indirectness, imprecision, and other considerations about publication bias, degree of the effect, presence of confounding factors, and dose-response gradient.

## 3. Results

### 3.1. Study Selection

**Figure 1:**
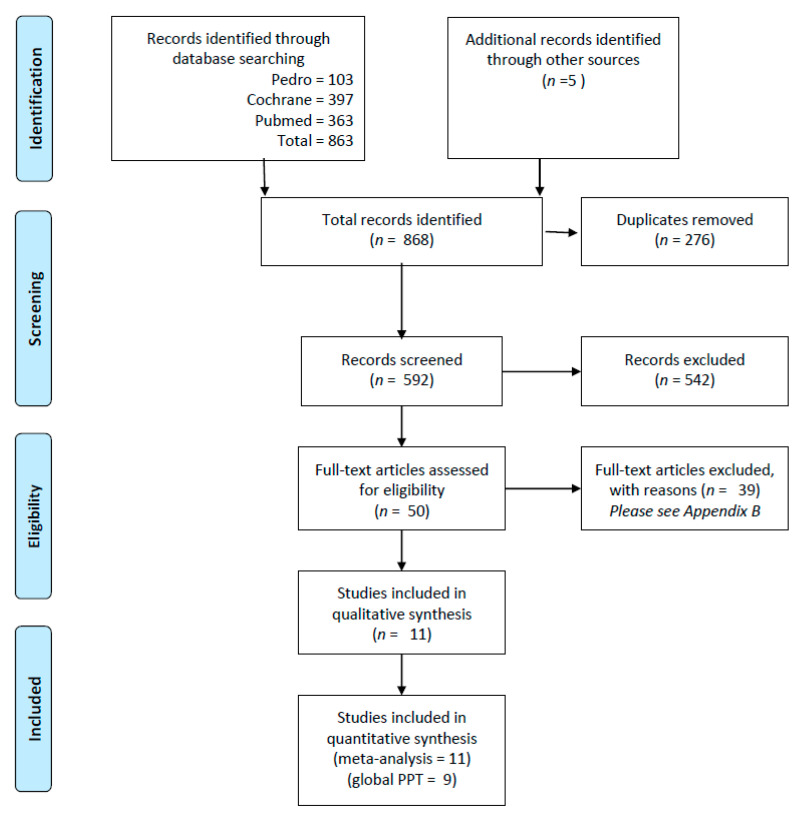
PRISMA flowchart of the systematic literature review [42].

The search strategy identified 868 records, of which 276 were duplicates. Following the screening process, 542 records were excluded. Of the 50 eligible articles, 39 were excluded after careful reading of the full-text, resulting in 11 articles included in the review [32,33,34,37,38,45,46,47,48,49,50]. The list of excluded articles and the reason for their exclusions are reported in Appendix B. Two additional articles [37,38] were discarded from the analysis of global PPT values for TrPs for reasons explained below. However, they were included in the comparisons regarding treatment effectiveness as the device used was the same for both the experimental and control group. The flowchart is reported in Figure 1. The inter-rater reliability was acceptable for the screening (k = 0.62, PA = 95.1%) and eligibility processes (k = 0.72, PA = 90%).

### 3.2. Study Characteristics

The majority of studies had an RCT design investigating the effect of various instrumental [34,38,48,49,50] or manual [32,33,34,46] physical therapy treatments on various clinical outcomes comprising the PPT (Table 1). The study by Abbaszadeh-Amirdehi et al. [45] was a prospective clinical trial and one study did not clearly report on the research design [47].

A total of 574 subjects (at least 309 female, as Abu Taleb et al. [32] and Öztürk et al. [50] did not report gender distribution), with a mean age of (29.64 ± 12.59 SD) years constituted the pooled population of subjects with TrPs. Two studies [34,46] also recruited a sample of 68 healthy controls (years, 36.1 ± 14.9 SD) to compare the PPT of the upper trapezius muscle without a TrP with the PPT of an upper trapezius hosting an active TrP of 104 subjects (years, 39.7 ± 12.3 SD).

Among the intervention studies, six studies investigating active TrPs recruited subjects with acute [32,37,38,49] or chronic [46,50] neck pain, while one study recruited subjects with myofascial pain syndrome [34] and one with shoulder pain [48]. One study recruited a pain-free population to investigate the PPT on latent TrPs [46], using parts of the clinical criteria, such as palpable taut band, hypersensitive tender spot, elicitation of local twitch response, and reproduction of referred pain pattern typical of the investigated muscle, used for the identification of active TrPs [1].

Six studies compared various manual techniques, comprising ischemic compression [32,37,46], algometer-driven ischemic compression [32], cervical spinal manipulation [46] or mobilization [46], active or passive positional release therapy [33], and pressure massage [34] with sham treatments (usually sham ultrasound [32,34] or sham procedure of the same therapy [33,37,46,47]) considered as placebos. The pooled population of the studies using manual intervention was composed of 310 participants (years, 26.52 ± 11.06 SD).

The studies comparing physical therapy modalities with the sham procedures used extracorporeal shockwave [48], ultrasound [34,38,49], low-level laser therapy [49], and kinesiotape [50] as interventions, while the same interventions with the device turned off or depowered were used in the placebo control groups of all these studies. The pooled population of this subgroup was constituted of 192 participants (years, 31.18 ± 11.92 SD).

Several types of algometers were employed across studies, of which only four precisely reported the instrument used [34,48,49,50]. Two studies [37,38] that neither described the algometer used nor reported the PPT measurement procedure (Table 2)were not included in calculating global PPT values for the PPT. Most studies used PPT values coming from the average of three repetitions at different time intervals lasting no longer than 60 s. The application rate of the algometer pressure was heterogeneous across studies. The PPT was measured after one treatment session in all the studies using a manual intervention, except for one study that measured the PPT after 24 h [46] and after three treatment sessions [33]. In the studies using instrumental physical therapy, the PPT was registered at the end of the treatments provided, which varied in number and frequency of sessions and lasted for 1–3 weeks (Table 2).

All the included studies referred to the diagnostic criteria [1] for the identification of either an active [33,37,44,47,48,49,51,52] or latent [32,45,46] TrP. An experienced practitioner made the diagnosis in seven studies [32,37,45,46,47,48], while five studies did not specify the experience of the examier. Only five studies [33,44,49,51,52] reported the location of the TrP in the upper trapezius and, consequently, the site of measurement (Table 3).

### 3.3. Risk of Bias within Studies

**Figure 2:**
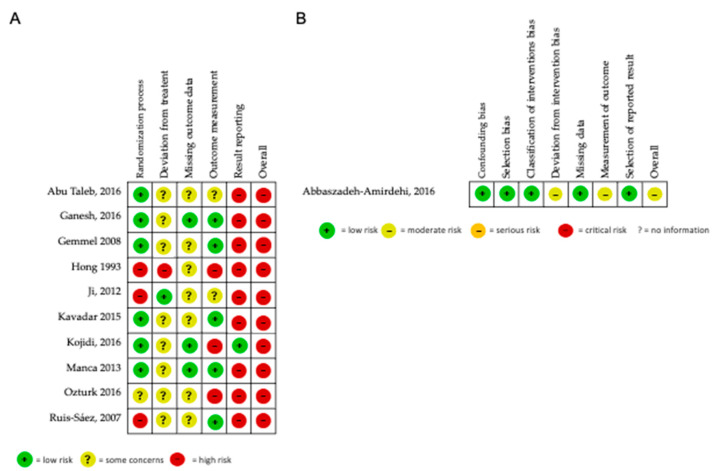
Risk of bias of the included studies. (**A**) RoB2 [51]; (**B**) ROBIN-I [31].

The risk-of-bias summary is reported in Figure 2A,B. All the studies were deemed as having a high risk of bias, as they presented at least one domain with a high risk of bias.

Among all the studies, one [34] had four domains with a high risk of bias. One non-randomized clinical trial [45] evaluated with ROBINS-I resulted in two domains with a moderate risk of bias. Three studies [47,48,50] had two domains with a high risk of bias, and six studies [32,33,37,38,46,49] had one domain with a high risk of bias. Among all domains, the one with a high risk of bias in the majority of the studies was the domain regarding results reporting [32,34,37,38,49,50], followed by the domain regarding the randomization process [34,45,47,48], measurement of the outcome [33,34,45,50], and deviation from the intended intervention [34,45]. The only domain without a high risk of bias in any study was the one regarding missing outcome data, that, on the other hand, showed concerns about its biasing in seven studies [32,34,37,38,47,48,50]. The inter-rater reliability of the risk of bias assessment was acceptable (k = 0.89, PA = 92.7%).

### 3.4. Synthesis of Results

**Figure 3:**
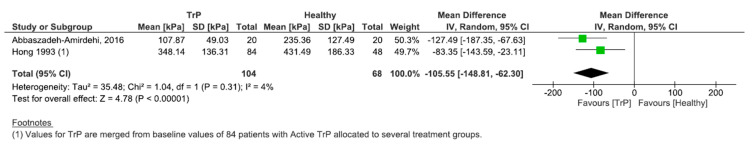
Forest plot for the comparison between patients with active TrPs and healthy controls.

Two studies (148 subjects) were meta-analyzed for the comparison between patients with active TrPs and healthy controls (Figure 3). The PPT was 105.55 kPa (95% CI: −148.81; −62.30) lower at active TrP sites (χ^2^ = 1.04, df = 1 (*p*= 0.31), I^2^ = 4%).

The weighted mean of the baseline PPT values coming from patients with active or latent TrPs and from the general healthy population gave overall values for these conditions (Table 4). The weighted mean of the PPT from the general population was 302.25 ± 36.94 kPa. One sample t-test showed lower values of the PPT for both active (weighted mean = −41.19 kPa, 95% CI: −53.77; −28.62; t = −6.44, df = 281, *p* < 0.001) and latent TrPs (weighted mean = −153.34, 95% CI: −156.59; −149.92; t = −88.8, df = 724, *p* < 0.001). In addition, a latent TrP hada lower PPT than an active TrP (weighted mean = −112.14 kPa, 95% CI: −99.61; −124.68), t = 17.613, df = 277, *p* < 0.001).

**Figure 4:**
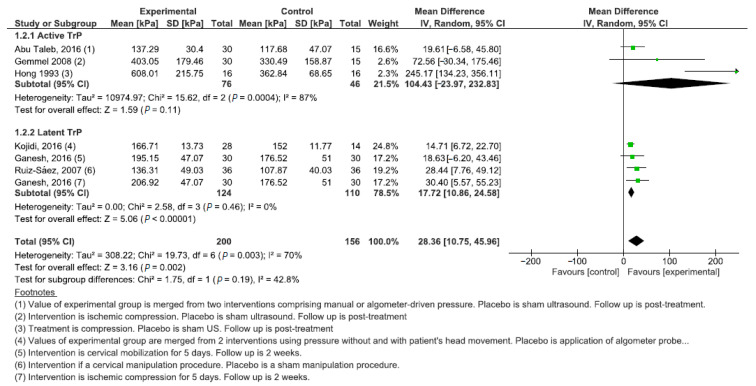
Forest plot for the comparison between manual intervention and minimal active treatment for active and latent TrPs.

**Figure 5:**
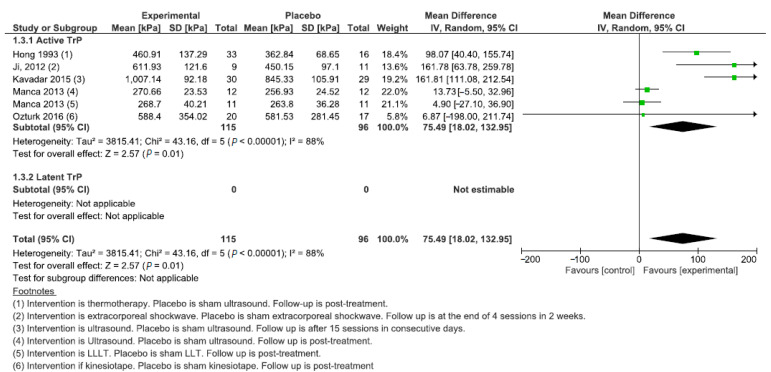
Forest plot for the comparison between physical therapy modalities and minimal active treatment.

Six studies (356 subjects) were meta-analyzed for the comparison between manual intervention and minimal active treatment with separate analyses for subgroups having active or latent TrPs. In general, the manual treatment effectively increased the PPT with an MD of 28.36 kPa (95% CI: 10.75; 45.96, χ^2^ = 19.73, df = 6 (*p* = 0.003), I^2^ = 70%). However, this positive result was biased by the subgroup with latent TrPs, while the separated analysis for the subgroup with active TrPs showed a large confidence interval with no effectiveness (PPT = 104.43 kPa; 95% CI: −23.97; 232.83) (Figure 4). The comparison between physical therapy modalities and minimal active treatment included six studies investigating active TrPs. The treatment effect was positive, with a PPT increase of 75.49 kPa (95% CI: 18.02; 132.95, χ^2^ = 43.16, df = 5 (*p* < 0.001), I^2^ = 88%) (Figure 5).

The summary of findings for each comparison and the quality of assessments are reported in Table 5.

### 3.5. Risk of Bias across Studies

**Figure 6:**
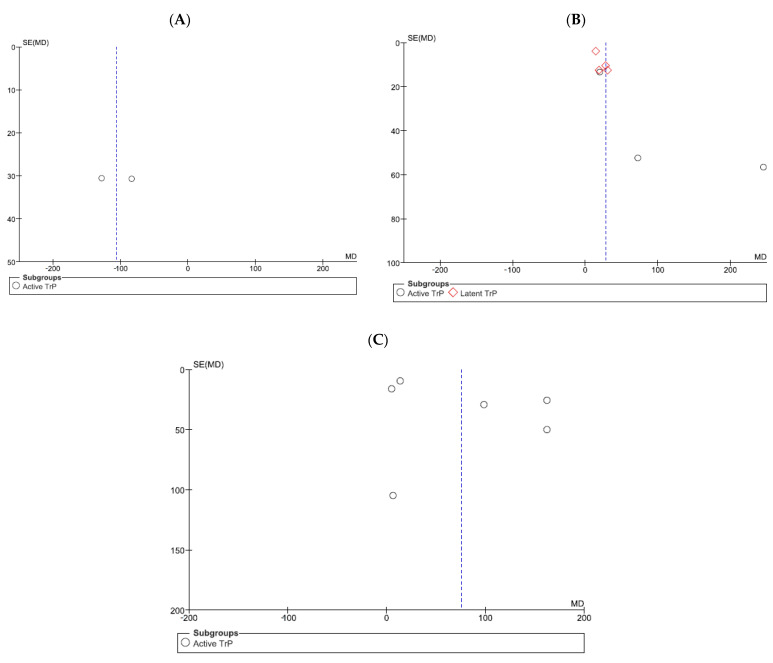
Funnel plot for the comparison between (**A**) patients with active TrPs and healthy controls, (**B**) manual treatment and minimal active intervention, and (**C**) physical therapy modalities and minimal active treatment.

A publication bias was observed for the comparison between manual treatment and minimal active intervention regarding the active TrP subgroup analysis (Figure 6b). Indeed, the distribution of studies on active TrPs was uneven across the pooled values, with two studies biasing the positive results of the meta-analysis. All the other comparisons did not show evidence of a publication bias as studies were evenly distributed among the pooled values (Figure 6).

## 4. Discussion

The main finding of this systematic review with meta-analysis was that the PPT was lower at active TrP sites of the upper trapezius when compared to the upper trapezius without TrPs of healthy subjects. The quality of the evidence was moderate, according to the GRADE tool. In all the retrieved studies [32,33,34,37,38,45,46,47,48,49,50], the TrP was first identified through manual palpation, and then the PPT over the TrP site was measured. Considering that the measurement of the PPT for active and latent TrPs comes from a young population with no difference in gender distribution, the results of the present review point out that the PPT values for TrPs were lower than the reference PPT values of the upper trapezius measured in two studies on pain-free populations with similar demographic characteristics [20,21]. Both these studies suggested the fifth percentile of the distribution to label hypersensitivity that can be roughly indicated as lower than 110 kPa [20] and 134 kPa [21]. For active TrPs, the only studies that yielded these criteria were those from Abbaszadeh-Amirdehi et al. [45] and Abu Taleb et al. [32] for active TrPs and Ruiz-Sáez et al. [47] for latent TrPs. As hypersensitivity of the trigger spot is considered a cardinal clinical criterion for the diagnosis of TrP [5], the use of different thresholds may affect the clinical assessment. When a patient’s complaints are driven by nociceptive pain and with signs that can be interpreted according to a peripheral sensitization of the pain system, then clinicians may refer to the normative PPT values reported in this review when comparing the affected upper trapezius with the contralateral one. Otherwise, when a patient’s complaints are compatible with a central sensitization syndrome, the comparison with the contralateral side may be inappropriate, with the risk of missing the decrease in PPT. In this particular case, the clinician should refer to the thresholds coming from studies on general pain-free populations [20,21]. Future studies may change the normative values reported in this review as only studies with a high risk of bias were included.

Another main finding was that either manual or physical therapy modalities and interventions are likely to increase the PPT values in subjects with active TrPs; however, it should be considered that the duration of the increase in PPT after an intervention has not been reported. The quality of the evidence was very low, according to the GRADE tool. The high heterogeneity observed in the comparisons between manual or instrumental treatment and minimal active intervention was expected as our main interest was in estimating the extent to which the PPT may vary following a treatment, rather than the effectiveness of the treatment itself, therefore studies with different clinical presentations, instruments and therapies used and their dosage, and the time to follow-up were inserted in the meta-analysis. The only criteria shared across all the retrieved studies was the use of established criteria [1] to diagnose a latent or an active TrP and a clear reporting of the algometer used. Both the analyzed interventions (manual or instrumental physical therapy treatment) showed an increase in PPT after physical or manual treatment; however, this result is likely to change as all the included studies were judged as having a high risk of bias. Furthermore, as the effectiveness of an intervention should consider thoroughly the patient’s health status instead of a mere modification of the PPT, we suggest that future studies should link the PPT change with change scores obtained through patient-reported outcome measures (e.g., disability, satisfaction) using a suitable analysis such as ROC curves.

Several hypotheses have been advanced to explain the somatosensory alterations caused by TrPs [53,54,55]. Despite controversies in the identification of the nociceptive locus, all point towards an increased afference to the motoneuron due to increased activity of the dorsal horn that may also explain the typical hypersensitivity found at TrP sites.

The fact that treatments were not as effective at restoring a normal PPT found in healthy subjects may partially explain why people do not recover fully after the first episode of neck pain [56]. It further supported the idea that once a TrP is treated (with manual or instrumental physical therapy treatment), a multimodal approach that also integrates exercises [57] is needed to favor the recovery of muscle that has developed a TrP. However, most of the studies included in this systematic review with meta-analysis measured the outcome of a single treatment approach in the short term (immediately after a one-session intervention). This fact should encourage clinicians and researchers to extend the follow-up to understand the influence of physical therapy interventions on the PPT of active TrPs in the long-term. In conclusion, measuring PPT values may constitute a valid procedure in supporting the diagnosis of myofascial TrPs in the upper trapezius muscle and monitoring a patient’s clinical improvement [15]. However, it should not be considered the first outcome measure as it relates only to a specific aspect of a multidimensional construct, such as a painful experience.

This study has some strengths and limitations that need consideration. The main strength of the study is that it is the first that has meta-analyzed PPT values in patients with active TrPs. This value, which had a moderate quality of evidence, may be used as a reference in clinical practice as well as in research in calculating the sample size for studies on the myofascial trigger point. Among the limitations, first, despite the search strategy encompassing three databases, some articles potentially affecting the result of the present review may have been retrieved with more databases included. Furthermore, only articles published in English were included in the review and this, as well as the lack of searching in the grey literature, may limit the generalizability of the result. Furthermore, all the analyzed studies had a high risk of bias, and a publication bias was found in the comparisonof manual and minimal active interventions. Moreover, the comparison of the PPT value between patients with an active TrP and healthy controls emerged from studies with different designs (one non-randomized clinical trial [46] and one RCT [35]). However, we only used the baseline values of the PPT; therefore, the different study designs should not affect the datum. Finally, the studies included in the meta-analysis were very heterogeneous in terms of the interventions and modalities; this may represent another limitation, as well as the heterogeneity of the duration of the treatment (some just one session, some multiple sessions). Therefore, future reviews on a similar topic based on more rigorous studies and also including studies with negative results may alter the reported findings.

## 5. Conclusions

Our findings showed that TrP has a decreased PPT when compared to an upper trapezius without TrP and healthy subjects and that either manual or physical therapy interventions may increase the PPT. However, the high risk of bias in all the included studies undermines the validity of the results.

## Figures and Tables

**Table 1:** Study characteristics.

Study	Design	Population	Number (Females)	Age (Years ± SD)	PPT Baseline (kPa)	Intervention
Abbaszadeh-Amirdehi et al. [45]	Prospective clinical trial	Persistent neck pain for more than 6 months Active TrP	20 [17]	31.7 ±10.9	107.87 ±49.03	Dry needling, one session, 3–5 times pistoning LTR elicitation not searched
		Healthy	20 [16]	30.4 ± 15.9	235.36 ± 127.49	Dry needling, one session, 3–5 times pistoning LTR elicitation not searched
Abu Taleb et al. [32]	RCT	Local pain in the upper trapezius area for no more than 12 weeks’ duration Active TrP	15	22.3 ± 3.8	109.83 ± 22.55	Algometer pressure release, 1 session, repeated 3 times at 30 s interval + sham ultrasound
			15	23.4 ± 5.1	133.37 ± 31.38	Manual pressure release, 1 session, repeated 3 times at 30 s interval + sham ultrasound
			15	22.8 ± 2.7	131.41 ± 40.21	Sham ultrasound, 1 session of 2 min
Ganesh et al. [46]	RCT	Cervical dysfunction ipsilateral to the TrP Latent TrP Decreased cervical contralateral flexion	30 [16]	22.03 ± 1.03	162.79 ± 50.01	C3–C4 PA mobilization (grade III-IV, rate 0.3 Hz, 4 repetitions), 1 session of 30 s repeated 3–4 times. Rest period: 1 min. 5 sessions in 5 days
			30 [17]	22.06 ± 1.08	172.6 ± 49.03	Ischemic compression, 1 session of 5–15 s repeated 4 times. 5 sessions in 5 days
			30 [21]	22.1 ± 1.04	161.81 ± 49.03	Sham procedure
Gemmell et al. [37]	RCT	Mechanical neck pain for less than 3 months Active TrP Pain VAS > 3 Decreased cervical contralateral flexion	15	24 ± 3.3	332.44 ± 113.76	Ischemic compression, 1 session, 60 s
			15	24 ± 4.6	274.59 ± 117.68	TrP Pressure Release, 1 session, 90 s
			15	23 ± 1.5	254.97 ± 81.39	Sham ultrasound, 1 session, 2 min
Hong et al. [34]	RCT	Myofascial pain syndrome (minimum pain duration, 8 months) Active TrP	16 [9]	42.5 ± 10.2	343.23 ± 88.25	Sham ultrasound, 1 session, 5 min, 0 W/cm^2^
			19 [11]	41.9 ± 9.2	343.23 ± 176.52	Spray and stretch, 1 session
			17 [10]	40.9 ± 8.9	333.43 ± 107.87	Hydrocollator, 1 session, 20–30min
			16 [10]	40.6 ± 9.2	372.65 ± 147.1	Ultrasound, 1 session, 5 min, 1.2–1.5 W/cm^2^
			16 [10]	432.6 ± 10	353.04 ± 127.49	Massage, 1 session, 10–15min, ischemic compression-like technique
		Healthy	24 [13]	40.8 ± 10	431.49 ±186.33	No treatment
Ji et al. [48]	RCT	Shoulder pain	9 [8]	32.82 ± 12.71	403.05 ± 99.05	Extracorporeal shock wave therapy, 1 session, 700 impulses to the taut band, 300 ipulses to the surrounding area, applied energy 0.056 mJ/mm^2^. 2 sessions per week in 2 weeks
			11 [9]	34 ± 15.56	436.4 ± 102.97	Extracorporeal shock wave therapy, 1 session, 700 impulses to the taut band, 300 impulses to the surrounding area, applied energy 0.001 mJ/mm^2^. 2 sessions per week in 2 weeks
Kavadar et al. [38]	RCT	Neck and/or back pain for no more than 6 weeks Active TrP	30 [24]	37.43 ± 9.07	725.69 ± 98.07	Ultrasound, 1 session, 6 min continuous mode with a dosage of 1.5 W/cm^2^ and 1 MHz frequency. 15 sessions
			29 [25]	35.83 ± 5.68	757.07 ± 104.93	Ultrasound, 1 session, 6 min with the device turned off. 15 sessions
Kojidi et al. [33]	RCT	Latent TrP Hypersensitive spot at 2.5 kg/cm^2^ of pressure	14 [14]	28.07 ± 6.24 (overall for 42 subjects)	148.08 ± 10.79	Active soft tissue therapy, 1 session, 3 repetitions, 20 s at 15 s interval. 3 sessions in 1 week
			14 [14]	/	151.02 ± 10.79	Passive soft tissue therapy, 1 session, 3 repetitions, 90 s at 15 s interval. 3 sessions in 1 week
			14 [14]	/	147.1 ± 12.74	Sham manual treatment, 1 session, 3 repetitions, 60 s at 15 s interval. 3 sessions in 1 week
Manca et al. [49]	RCT	Spontaneous pain and palpable taut band in upper trapezius disturbing normal daily activity	12 [7]	24.5 ± 1.7	208.88 ± 23.53	Ultrasound, one session of 12 min continuous mode with a dosage of 1.5 W/cm^2^ and 3 MHz frequency. 10 sessions in 2 weeks
			12 [6]	26 ± 0.8	208.88 ± 19.61	Sham ultrasound, 1 session, 12 min with the device turned off. 10 sessions in 2 weeks
			11 [7]	24 ± 2.1	198.09 ± 33.34	Low-level laser therapy, 1 session, 10 min, energy of 18 J. 10 sessions in 2 weeks.
			11 [7]	25.4 ± 0.7	204.96 ± 26.48	Sham low-level laser therapy, 1 session, 10 min with the device turned off.
			14 [7]	23 ± 1.91	207.9 ± 19.6	No intervention
Öztürk et al. [50]	RCT	Neck or back pain for more than 2 weeks Active TrP	20	22.95 ± 4.9	377.56 ± 256.93	Kinesiotape, 1 application, 3 days. 2 applications in 1 week.
			17	33.86 ± 8.47	483.47 ± 248.11	Sham-kinesiotape, 1 application, 3 days. 2 applications in 1 week
Ruiz-Sáez et al. [47]	NR	Pain-free population Latent TrP Intervertebral C3–C4 joint dysfunction ipsilateral to the TrP	36 [22]	31 ± 7	124.54 ± 49.03	C3-C4 HVLAT, 1 session
			36 [24]	32 ± 11	131.41 ± 39.23	Sham procedure, 1 session, 30 s

Legend: HVLAT, High Velocity Low Amplitude Thrust; kPa, kilopascal; LTR, Local Twitch Response; NR, Not Reported; PA, Posterior-to-Anterior; PPT, Pressure Pain Threshold; RCT, Randomized Controlled Trial; SD, Standard Deviation; TrP, Trigger Point; VAS, Visual Analogue Scale.

**Table 2:** Characteristics of algometry and PPT values among subgroups.

Study	Algometer Device and Company	PPT Measure	Follow-Up	PPT Intervention (kPa) Mean ± SD	PPT Control (kPa) Mean ± SD
Abbaszadeh-Amirdehi et al. [45]	NR Digital Instrument, Lutron, Taiwan	Average of 3 times at 40 s interval	Post-treatment	107.87 ± 49.03	235.36 ± 127.49
Abu Taleb et al. [32]	NR Wagner Instrument, CT, USA	Average of 3 times	Post-treatment	137.29 ± 30.4	117.68 ± 47.07
Ganesh et al. [46]	NR Electronic Engineering Corporation, Chennai, India	Average of 3 times at 30 s interval	After 24 h	206.92 ± 47.07 195.15 ± 47.07	176.52 ± 51
Gemmell et al. [37]	NR	NR	Post-treatment	403.05 ± 179.46	330.48 ± 158.87
Hong et al. [34]	Pressure threshold meter Pain Diagnostic and Thermography, Great Neck, NY, USA	Average of 3 times at 20–60 s interval	Post-treatment	348.13 ± 136.31	431.49 ± 186.33
Ji et al. [48]	OE-220^®^ ITO., Tokyo, Japan	Average of 3 times at 10 s interval	After 4 sessions in 3 weeks	611.93 ± 121.6	450.15 ± 97.1
Kavadar et al. [38]	NR	Average of 3 times at 60 s interval	After 15 sessions	1007.14 ± 92.18	845.33 ± 105.91
Kojidi et al. [33]	5020 version Taiwan	NR	After 3 sessions	166.71 ± 13.73	152 ± 11.77
Manca et al. [49]	FDK-20 Wagner Instruments, Greenwich, CT, USA	Average of 3 times at 20 s interval	After 10 sessions in 2 weeks	270.66 ± 23.53 268.7 ± 40.21	256.93 ± 24.52 263.8 ± 36.28
Öztürk et al. [50]	FDK Wagner Instruments, Riverside, CT, USA	Average of 3 times at 60 s interval	After 2 applications in 1 week	588.4 ± 354.02	581.53 ± 281.45
Ruiz-Sáez et al. [47]	- Pain Diagnosis and Treatment Inc, Great Neck, NY, USA	Average of 3 times at 30 s interval	After 10 min	136.31 ± 49.03	107.87 ± 40.03

Legend: kPa, kiloPascal; NR, Not Reported; PPT, Pressure Pain Threshold; SD, Standard Deviation.

**Table 3:** Characteristics of examination of the upper trapezius muscle.

Study	Examiner Characteristic	TrP Type	Specified Location	Specified Criteria According to Simons et al. [1]
Abbaszadeh-Amirdehi et al. [45]	NR	Active	X	X
Abu Taleb et al. [32]	NR	Active		X
Ganesh et al. [46]	More than 10 years of clinical experience in diagnosingTrP	Latent		X
Gemmell et al. [37]	NR	Active	X	X
Hong et al. [34]	NR	Active	X	X
Ji et al. [48]	Medical doctor	Active		X
Kavadar et al. [38]	Physician	Active		X
Kojidi et al. [33]	Physiotherapy student with 6 years of university study	Latent		X
Manca et al. [49]	Orthopedic physician experienced in musculoskeletal disorders	Active	X	X
Öztürk et al. [50]	NR	Active		X
Ruiz-Sáez et al. [47]	Physiotherapist with 4 years or more of clinical experience in diagnosing TrP	Latent		X

Legend: NR, Not Reported; TrP, Trigger Point.

**Table 4:** Results for active and latent trigger points.

TrP Condition	Study	N	PPT ±SD (kPa)
Active	Abbaszadeh-Amirdehi et al. [45]	20	107.87 ± 40.03
	Abu Taleb et al. [32]	45	124.87 ± 37.27
	Gemmell et al. [37]	45	287.33 ± 108.34
	Hong et al. [34]	84	348.72 ± 132.02
	Ji et al. [48]	20	421.39 ± 104.87
	Manca et al. [49]	46	205.36 ± 25.53
	Overall	260	261.05 ± 100.91
Latent	Ganesh et al. [46]	90	165.73 ±49.05
	Kojidi et al. [33]	42	148.73 ± 11.51
	Ruiz-Sáez et al. [47]	72	127.98 ± 44.22
	Overall	204	148.91 ± 16.75
Pain-free population	Neziri et al. [20]	150	262.5 ± 98.36
	Waller et al. [21]	611	304.04 ± 177.07
	Overall		302.25 ± 36.94

**Table 5:** Summary of findings for the comparisons.

Certainty Assessment							No of Patients		Effect		Certainty	Importance
No of Studies	Study Design	Risk of Bias	Inconsistency	Indirectness	Imprecision	Other Considerations	Manual Treatment	Other Treatments	Relative (95% CI)	Absolute (95% CI)		
Active trigger point vs. Healthy controls												
2	randomised trials	very serious	not serious	not serious	not serious	strong association	104	68	-	MD 105.55 lower (148.81 lower to 62.3 lower)	⨁⨁⨁◯Moderate	CRITICAL
Manual treatment vs. Minimal active intervention												
6	randomised trials	very serious	serious ^a^	not serious	very serious ^a^	publication bias strongly suspected ^b^	200	156	-	MD 28.36 higher (10.75 higher to 45.96 higher)	⨁◯◯◯Very low	IMPORTANT
Physical therapy modalities vs. Minimal active intervention												
5	randomised trials	very serious	serious ^c^	not serious	very serious ^c^	none	115	96	-	MD 75.49 higher (18.02 higher to 132.95 higher)	⨁◯◯◯Very low	IMPORTANT

CI: Confidence Interval; MD: Mean Difference. Notes. a. Several physical therapy modalities have been used. b. The funnel plot is skewed towards studies with positive results. c. Several types of manual treatment have been used.

## Data Availability

Not applicable.

## Appendix A

**Table A1:** Search strategy.

Electronic Database	Search String
PubMed	((((“pressure pain threshold”)) OR (algometer)) OR (algometry))) AND ((((((“trigger points”)) OR (“trigger point”)) OR (“myofascial pain”)) OR (“Trigger Points”[Mesh])) OR (“Myofascial Pain Syndromes”[Mesh]))
PEDro	pressure pain threshold AND trigger point*
Cochrane Library	((((“pressure pain threshold”)) OR (algometer)) OR (algometry))) AND ((((((“trigger points”)) OR (“trigger point”)) OR (“myofascial pain”))

## Appendix B

**Table A2:** List of excluded studies after full text reading with reasons for exclusion.

Number	Reference	Reason for Exclusion
1	Amini A, Goljaryan S, Shakouri SK, Mohammadimajd E. The Effects of Manual Passive Muscle Shortening and Positional Release Therapy on Latent Myofascial Trigger Points of the Upper Trapezius: A Double-Blind Randomized Clinical Trial. Iranian Red Crescent Medical Journal, 2017, 19.9.	The study compared two manual interventions without a control group
2	Ay S, Doğan SK, Evcik D, Başer OC. Comparison the efficacy of phonophoresis and ultrasound therapy in myofascial pain syndrome. Rheumatol Int. 2011 Sep;31(9):1203-8. doi: 10.1007/s00296-010-1419-0.	The study provided PPT values in Newton without area of application (nokg/cm^2^)
3	Ay S, Konak HE, Evcik D, Kibar S. The effectiveness of Kinesio Taping on pain and disability in cervical myofascial pain syndrome. Rev Bras Reumatol Engl Ed. 2017 Mar-Apr;57(2):93-99 doi: 10.1016/j.rbre.2016.03.012.	The study provided PPT values in Newton without area of application (nokg/cm^2^)
4	Bae Y. Change the myofascial pain and range of motion of the temporomandibular joint following kinesio taping of latent myofascial trigger points in the sternocleidomastoid muscle. J Phys Ther Sci. 2014 Sep;26(9):1321-4. doi: 10.1589/jpts.26.1321.	The PPT measurement in the study is not related to upper trapezius muscles
5	Bahadır C, Dayan VY, Ocak F, Yiğit S. Efficacy of immediate rewarming with moist heat after conventional vapocoolant spray therapy in myofascial pain syndrome. Journal of Musculoskeletal Pain, 2010, 18.2: 147-152.	The study compared two physical therapy modality interventions without acontrol group
6	Bakar Y, Sertel M, Oztürk A, Yümin ET, Tatarli N, Ankarali H. Short term effects of classic massage compared to connective tissue massage on pressure pain threshold and muscle relaxation response in women with chronic neck pain: a preliminary study. J Manipulative Physiol Ther. 2014 Jul-Aug;37(6):415-21. doi: 10.1016/j.jmpt.2014.05.004.	The study compared two interventions without a control group
7	Benjaboonyanupap D, Paungmali A, Pirunsan U. Effect of Therapeutic Sequence of Hot Pack and Ultrasound on Physiological Response Over Trigger Point of Upper Trapezius. Asian J Sports Med. 2015 Sep;6(3):e23806. doi: 10.5812/asjsm.23806.	The study compared two manualinterventions without a control group
8	Boonruab J, Damjuti W, Niempoog S, Pattaraarchachai J. Effectiveness of hot herbal compress versus topical diclofenac in treating patients with myofascial pain syndrome. J Tradit Complement Med. 2018 Jun 1;9(2):163-167. doi: 10.1016/j.jtcme.2018.05.004.	The study compared two interventions without a control group
9	Cerezo-Téllez E, Lacomba MT, Fuentes-Gallardo I, Mayoral Del Moral O, Rodrigo-Medina B, Gutiérrez Ortega C. Dry needling of the trapezius muscle in office workers with neck pain: a randomized clinical trial. J Man Manip Ther. 2016 Sep;24(4):223-32. doi: 10.1179/2042618615Y.0000000004.	The study compared two interventions without a control group
10	Chao YW, Lin JJ, Yang JL, Wang WT. Kinesio taping and manual pressure release: Short-term effects in subjects with myofasical trigger point. J Hand Ther. 2016 Jan-Mar;29(1):23-9. doi: 10.1016/j.jht.2015.10.003.	The study compared two interventions without a control group
11	Chou LW, Hsieh YL, Chen HS, Hong CZ, Kao MJ, Han TI. Remote therapeutic effectiveness of acupuncture in treating myofascial trigger point of the upper trapezius muscle. Am J Phys Med Rehabil. 2011 Dec;90(12):1036-49. doi: 10.1097/PHM.0b013e3182328875	The intervention is not directed to the PPT measured muscle
12	Dibai-Filho AV, de Oliveira AK, Girasol CE, Dias FR, Guirro RR. Additional Effect of Static Ultrasound and Diadynamic Currents on Myofascial Trigger Points in a Manual Therapy Program for Patients With Chronic Neck Pain: A Randomized Clinical Trial. Am J Phys Med Rehabil. 2017 Apr;96(4):243-252. doi: 10.1097/PHM.0000000000000595.	The study compared two manual interventions without a control group
13	Dıraçoğlu D, Vural M, Karan A, Aksoy C. Effectiveness of dry needling for the treatment of temporomandibular myofascial pain: a double-blind, randomized, placebo controlled study. J Back Musculoskelet Rehabil. 2012;25(4):285-90. doi: 10.3233/BMR-2012-0338.	The PPT measurement in the study is not related to upper trapezius muscles
14	Edwards J, Knowles N. Superficial dry needling and active stretching in the treatment of myofascial pain--a randomised controlled trial. Acupunct Med. 2003 Sep;21(3):80-6. doi: 10.1136/aim.21.3.80.	The PPT measurement in the study is not related to upper trapezius muscles
15	Fernández-Carnero J, Gilarranz-de-Frutos L, León-Hernández JV, Pecos-Martin D, Alguacil-Diego I, Gallego-Izquierdo T, Martín-Pintado-Zugasti A. Effectiveness of Different Deep Dry Needling Dosages in the Treatment of Patients With Cervical Myofascial Pain: A Pilot RCT. Am J Phys Med Rehabil. 2017 Oct;96(10):726-733. doi: 10.1097/PHM.0000000000000733.	The study is about the change in LTR after different depth of insertion
16	Gemmell H, Allen A. Relative immediate effect of ischaemic compression and activator trigger point therapy on active upper trapezius trigger points: A randomised trial. Clinical Chiropractic, 2008, 11.4: 175-181.	The study compared two manual interventions without a control group
17	Gemmell H, Hilland A. Immediate effect of electric point stimulation (TENS) in treating latent upper trapezius trigger points: a double blind randomised placebo-controlled trial. J Bodyw Mov Ther. 2011 Jul;15(3):348-54. doi: 10.1016/j.jbmt.2010.04.003.	The study did not provide the raw post-intervention values but only thewithin-group pre-post differences
18	Gordon CM, Andrasik F, Schleip R, Birbaumer N, Rea M. Myofascial triggerpoint release (MTR) for treating chronic shoulder pain: A novel approach. J Bodyw Mov Ther. 2016 Jul;20(3):614-22. doi: 10.1016/j.jbmt.2016.01.009.	The study did not provide PPT values in kg/cm^2^
19	Graff-Radford SB, Reeves JL, Baker RL, Chiu D. Effects of transcutaneous electrical nerve stimulation on myofascial pain and trigger point sensitivity. Pain. 1989 Apr;37(1):1-5. doi: 10.1016/0304-3959(89)90146-2.	The study merged values from several muscles
20	Gulick DT, Palombaro K, Lattanzi JB. Effect of ischemic pressure using a Backnobber II device on discomfort associated with myofascial trigger points. J Bodyw Mov Ther. 2011 Jul;15(3):319-25. doi: 10.1016/j.jbmt.2010.06.007.	The study did not specify the TrP that are measured or treated
21	Hakim IK, Takamjani IE, Sarrafzadeh J, Ezzati K, Bagheri R. The effect of dry needling on the active trigger point of upper trapezius muscle: Eliciting local twitch response on long-term clinical outcomes. J Back Musculoskelet Rehabil. 2019;32(5):717-724. doi: 10.3233/BMR-181286.	The study is about the difference in diagnostic criteria (LTR present or not) inthe effectiveness of DN
22	Ibáñez-García J, Alburquerque-Sendín F, Rodríguez-Blanco C, Girao D, Atienza-Meseguer A, Planella-Abella S, Fernández-de-Las Peñas C. Changes in masseter muscle trigger points following strain-counterstrain or neuro-muscular technique. J Bodyw Mov Ther. 2009 Jan;13(1):2-10. doi: 10.1016/j.jbmt.2008.03.001.	The PPT measurement in the study is not related to upper trapezius muscles
23	Jaeger B, Reeves JL. Quantification of changes in myofascial trigger point sensitivity with the pressure algometer following passive stretch. Pain. 1986 Nov;27(2):203-210. doi: 10.1016/0304-3959(86)90211-3.	The study merge values from both upper trapezius and elevator scapula muscles
24	Mohammadi Kojidi M, Okhovatian F, Rahimi A, Baghban AA, Azimi H. The influence of Positional Release Therapy on the myofascial trigger points of the upper trapezius muscle in computer users. J Bodyw Mov Ther. 2016 Oct;20(4):767-773. doi: 10.1016/j.jbmt.2016.04.006.	Data are the same reported in Kojidi, 2016
25	Mohamadi M, Piroozi S, Rashidi I, Hosseinifard S. Friction massage versus kinesiotaping for short-term management of latent trigger points in the upper trapezius: a randomized controlled trial. Chiropr Man Therap. 2017 Sep 12;25:25. doi: 10.1186/s12998-017-0156-9	The study compared two manual interventions without a control group
26	Lai YT, Chan HL, Lin SH, Lin CC, Li SY, Liu CK, Teng HW, Liu WS. Far-infrared ray patches relieve pain and improve skin sensitivity in myofascial pain syndrome: A double-blind randomized controlled study. Complement Ther Med. 2017 Dec;35:127-132. doi: 10.1016/j.ctim.2017.10.007.	The study did not report clearly the population
27	Lee SH, Lu WA, Lee CS, Wang JC, Lin TC, Yang JL, Chan RC, Ko SC, Kuo CD. The therapeutic effect of collateral meridian therapy is comparable to acupoint pressure therapy in treating myofascial pain syndrome. Complement Ther Clin Pract. 2014 Nov;20(4):243-50. doi: 10.1016/j.ctcp.2014.10.003.	The study reported data as Median and IQR
28	Martín-Pintado-Zugasti A, Fernández-Carnero J, León-Hernández JV, Calvo-Lobo C, Beltran-Alacreu H, Alguacil-Diego I, Gallego-Izquierdo T, Pecos-Martin D. Postneedling Soreness and Tenderness After Different Dosages of Dry Needling of an Active Myofascial Trigger Point in Patients With Neck Pain: A Randomized Controlled Trial. PM R. 2018 Dec;10(12):1311-1320. doi: 10.1016/j.pmrj.2018.05.015.	The study is about the difference in soreness and other contraindication to DN(LTR present or not) after different type of DN
29	Moraska AF, Schmiege SJ, Mann JD, Butryn N, Krutsch JP. Responsiveness of Myofascial Trigger Points to Single and Multiple Trigger Point Release Massages: A Randomized, Placebo Controlled Trial. Am J Phys Med Rehabil. 2017 Sep;96(9):639-645. doi: 10.1097/PHM.0000000000000728.	The study merged PPT values from both active and latent trigger point
30	Oliveira-Campelo NM, Rubens-Rebelatto J, Martí N-Vallejo FJ, Alburquerque-Sendí N F, Fernández-de-Las-Peñas C. The immediate effects of atlanto-occipital joint manipulation and suboccipital muscle inhibition technique on active mouth opening and pressure pain sensitivity over latent myofascial trigger points in the masticatory muscles. J Orthop Sports Phys Ther. 2010 May;40(5):310-7. doi: 10.2519/jospt.2010.3257.	The intervention is not directed to the PPT measured muscle
31	Pecos-Martín D, Montañez-Aguilera FJ, Gallego-Izquierdo T, Urraca-Gesto A, Gómez-Conesa A, Romero-Franco N, Plaza-Manzano G. Effectiveness of dry needling on the lower trapezius in patients with mechanical neck pain: a randomized controlled trial. Arch Phys Med Rehabil. 2015 May;96(5):775-81. doi: 10.1016/j.apmr.2014.12.016.	The muscle is not upper trapezius
32	Pecos-Martin D, Ponce-Castro MJ, Jiménez-Rejano JJ, Nunez-Nagy S, Calvo-Lobo C, Gallego-Izquierdo T. Immediate effects of variable durations of pressure release technique on latent myofascial trigger points of the levator scapulae: a double-blinded randomised clinical trial. Acupunct Med. 2019 Jun;37(3):141-150. doi: 10.1136/acupmed-2018-011738.	The study compared three interventions without a control group
33	Shakeri H, Soleimanifar M, Arab AM, Hamneshin Behbahani S. The effects of KinesioTape on the treatment of lateral epicondylitis. J Hand Ther. 2018 Jan-Mar;31(1):35-41. doi: 10.1016/j.jht.2017.01.001.	The study did not reported the unit of measure used for PPT
34	Srbely JZ, Vernon H, Lee D, Polgar M. Immediate effects of spinal manipulative therapy on regional antinociceptive effects in myofascial tissues in healthy young adults. J Manipulative Physiol Ther. 2013 Jul-Aug;36(6):333-41. doi: 10.1016/j.jmpt.2013.01.005.	The study provided PPT values in Newton without area of application (NO kg/cm^2^)
35	Wilke J, Vogt L, Banzer W. Immediate effects of self-myofascial release on latent trigger point sensitivity: a randomized, placebo-controlled trial. Biol Sport. 2018 Dec;35(4):349-354. doi: 10.5114/biolsport.2018.78055. Epub 2018 Aug 31.	The muscle is not upper trapezius
36	Wilke J, Vogt L, Niederer D, Hübscher M, Rothmayr J, Ivkovic D, Rickert M, Banzer W. Short-term effects of acupuncture and stretching on myofascial trigger point pain of the neck: a blinded, placebo-controlled RCT. Complement Ther Med. 2014 Oct;22(5):835-41. doi: 10.1016/j.ctim.2014.09.001.	The study did not provide data at post-intervention but only graphs.
37	Wilke J, Niederer D, Fleckenstein J, Vogt L, Banzer W. Range of motion and cervical myofascial pain. J Bodyw Mov Ther. 2016 Jan;20(1):52-55. doi: 10.1016/j.jbmt.2015.04.003.	The study did not measure PPT for the healthy controls.
38	Wytrążek M, Huber J, Lipiec J, Kulczyk A. Evaluation of palpation, pressure algometry, and electromyography for monitoring trigger points in young participants. J Manipulative Physiol Ther. 2015 Mar-Apr;38(3):232-43. doi: 10.1016/j.jmpt.2014.12.005.	The study is on healthy population but active TrP are detected
